# Rural general practice staff experiences of patient safety incidents and low quality of care in Norway: an interview study

**DOI:** 10.1093/fampra/cmab064

**Published:** 2021-06-28

**Authors:** Martin B Harbitz, Per S Stensland, Margrete Gaski

**Affiliations:** 1 Department of Community Medicine, UiT The Arctic University of Norway, Norwegian Centre for Rural Medicine, Tromsø; 2 Department of Global Public Health and Primary Care, University of Bergen, Bergen; 3 Department of Community Medicine, UiT The Arctic University of Norway, Norwegian Centre for Rural Medicine, Tromsø, Norway

**Keywords:** General practitioners, interview, medical secretaries, patient safety, primary health care, rural health

## Abstract

**Background and objectives:**

General practitioners (GPs), nurses and medical secretaries (practice staff) are responsible for the continuous provision of safe care in rural general practice. Little is known about their role in situations where patients were or could have been harmed in a rural setting. Therefore, we sought to investigate rural general practice staff experiences of patient safety incidents and low quality of care.

**Methods:**

Descriptive qualitative interviews using the critical incident technique. Systematic text condensation analysis involving GPs and practice staff in eight rural municipalities in Norway.

**Results:**

Sixteen participants (eight GPs, one nurse and seven medical secretaries) with mean work experience of 11.8 years were interviewed for a total of 11.5 hours. We identified three main factors that make rural GP clinics vulnerable to patient safety incidents and low quality of care: use of locums, work overload and rough weather and distance to hospital. There was a wide range of patient safety incidents. The healthcare personnel explained how they used local knowledge about people and context and greater awareness of risk of error in order to prevent these incidents from happening.

**Conclusion:**

Rural GP clinics that suffer from frequent use of GP locums and work overload are vulnerable to patient safety incidents. Practice staff use various forms of continuity of care to prevent safety incidents from happening; this highlights the strengths but also some major safety concerns in these GP clinics. Staff at these clinics proved to be a resource for patient safety research.

**Podcast:**

An accompanying podcast on patient safety is available as Supplementary Data, in which Martin Bruusgaardf Harbitz and Per Stensland provide insights into the context of this study.

## Lay Summary

When we go to see the doctor, we all want our diagnosis and treatment to be safe and free from mistakes. Unfortunately, patient harm and low quality of care happen every day in medical practice. This article looks at staff experiences of these mistakes; the staff were general practitioners, nurses and medical secretaries. We show how the use of locum doctors, work overload and long distance to hospital are linked to examples of patient harm. Our findings also show how nurses and medical secretaries may help to prevent harm to patients.

**Key Messages**

• Patient safety in rural general practice needs attention.
• Qualitative interviews reveal troublesome issues.
• Frequent use of locums can impair patient safety in rural general practice.
• Irresponsible practice, lack of follow-up, low trust and low support were found.
• Nurses and secretaries can play an important role in promoting patient safety.

## Background

Medical secretaries and nurses (practice staff) are often the first healthcare personnel that people meet when seeking medical help at a primary care facility. Up to 90% of all healthcare contacts in Western societies take place in general practice (1) where it is estimated that 2–3% of the consultations include an unintended incident that resulted or could have resulted in patient harm—a patient safety incident (2,3). The associated emotional and financial costs are substantial (4–6). In a Norwegian population study, patients blamed the general practitioner (GP) after having experienced a patient safety incident (7). However, the literature reveals little research on the role and experience of GPs and other staff in this area.

All registered inhabitants of Norway are entitled to be on a GP’s list. Most of the treatment costs are covered by the state. In Norwegian GP clinics, authorized practice staff work together with the GPs. They support the GPs in clinical procedures and tests. The GPs are responsible for all the patients on their patient list; this is a systematic way of providing continuity of care, defined as the care of individuals over time. Continuity enhances GP knowledge about patients, which can increase patient trust and improve compliance (8). However, continuity varies across Norway. GPs’ median length of work experience is 2.8 years in municipalities with a small population, while the figure increases threefold for municipalities with a large population (9). In small municipalities, GP continuity is affected by frequent use of locums [substitute GPs] (9). In 2014, there were 67 GP lists without a regular GP. Over 52% of the locums working on these lists were recruited by agencies and did not have a permanent address in Norway, and 75% of the locums worked in rural municipalities (10). Today the number of GP lists without a regular GP is 182 (11), which gives rise to more and more ‘relays of locums’ profiting from fee-for-service schemes and replacing the stable regular GPs (12). The municipalities are responsible for hiring qualified locums and the government requires GPs to specialize in family medicine in order to practice; this is, however, not a requirement for locum GPs (13). Rural GPs receive 69% more disciplinary actions than urban GPs (14). Little is known about patient safety threats in these clinics, which calls for research in the area (8,15,16). The purpose of this article is to investigate rural general practice staff experiences of patient safety incidents and low quality of care, using critical incident technique (CIT) interviews.

## Methods

### Contributors

We chose a descriptive qualitative approach for our study, based on interviews and notes (17). To enhance validity and relevance (18), we interviewed GPs and practice staff working in rural GP clinics. Due to the ongoing COVID-19 pandemic, we had to redesign our study from meetings in person to Skype interviews. In cases of communication breakdown on Skype, we continued interviewing over the phone. M.B.H. and M.G. conducted the interviews and kept notes based on observations and reflections during interviews. All authors contributed to designing the study, analysing the material, interpreting and critically revising the manuscript. The interviews were digitally audiotaped and transcribed verbatim.

### Recruitment and participants

To capture the most prominent and typical safety issues in rural municipalities with challenging distance to hospital, we performed purposive sampling of experienced rural primary healthcare workers. Eight district medical officers in Northern Norway were contacted in March 2020 about the study and they all confirmed participation. The district medical officers recruited local GPs and practice staff with permanent positions and considerable work experience, locums were excluded. Sixteen participants agreed to participate, one declined because he was not currently doing clinical work. All of the GP clinics were located in rural municipalities, according to Statistics Norway. The interviewees signed a document on study aims and rights.

### Interview design and procedure

We chose to investigate clinicians’ behaviour and experiences with patient safety incidents through the ‘Critical Incident Technique’ (CIT) (19). Since its original development (20), CIT has proven useful in addressing tacit knowledge and actual performance in incidents occurring in hospitals (21) and general practice (15). We judged patient safety incidents to be critical incidents. We asked participants to prepare to describe a specific event where a patient was, or could have been, harmed. See Table 1 for interview questions. We did not seek saturation, although after 16 interviews we concluded that the dataset was consistent with the study aim.

**Table 1.** Interview questions used in the critical incident technique, March 2020

Interview guide	Follow-up questions
‘Can you tell me about the unsafe event when you were unable to prevent harm to the patient?’	*‘*How did the patient react? What were the consequences?*’* *‘*What was your emotional response?*’* *‘*Were there any circumstances that may have made this event happen?*’*
‘What were the professional and personal characteristics of the doctor or other healthcare personnel in this event?’	*‘*Have you had colleagues that you thought posed a risk to the patients’ safety?*’* *‘*Can you describe what this risk consisted of?*’*
‘What did the doctor or healthcare personnel do in this event?’	*‘*Is this something you repeatedly practice?*’*
‘Did you do anything to prevent the incident?’	*‘*What did you learn from the experience? *‘*How did you talk about this event with your colleagues?*’*
‘Are there any other typical situations you find risky for patient safety?’	*‘*What patient safety responsibility do local decision makers and administrative staff have?*’*

### Data analysis

Systematic text condensation is a pragmatic method (22) using cross-case analysis to develop new descriptions and concepts of phenomena based on perspectives on how they are experienced (23). See Table 2 for the analytic process. Participants’ statements were anonymized and assigned a random letter. We did not perform participant validation. The study followed the Consolidated Criteria for Reporting Qualitative Research (COREQ) checklist (24).

**Table 2.** The stepwise process of systematic text condensation

Steps in systematic text condensation (23)	Codes and themes
**Step 1:** Initially, all researchers read through all transcribed interviews and field notes, following the stepwise analysis process of systematic text condensation, starting with identifying preliminary themes.	1) risk characteristics, 2) medical errors, 3) actions to avoid harm, 4) coping with risk.
**Step 2:** Manually coding the interviews according to semantic content. This involved identifying meaning units associated with each theme, and temporarily removing parts of the text from their context (decontextualization) and sorting them into code groups.	
**Step 3:** By expressing the content of the meaning units across all participants under each code group we realized that our initial names of themes did not suitably cover our new code groups.	1) rural vulnerability, 2) fallible rural practice, 3) keeping rural practice safe.
**Step 4:** The fourth and final step involved reconceptualization of the data to make a synthesis of the condensates.	1) vulnerability in rural practice, 2) a wide range of patient safety incidents, 3) keeping the clinic safe.

## Results

### Participants

Table 3 presents descriptive data about the interviews, the participants and the context.

**Table 3.** Descriptive data of 16 rural GP staff who participated in the study, April 2020

	General practitioners	Nurses	Medical secretaries*	Total
Average clinical work experience	12.1 years		11.5 years	201 years
Number of participants	4 women 4 men	1 man	7 women	11 women 5 men
Type of interview	Skype: 3 Phone: 5	Skype: 1	Skype: 3 Phone: 4	Skype: 7 Phone: 9
Average interview duration	45.1 minutes	53 minutes	39.4 minutes	690 minutes
Average distance to hospital	By car on average 184 km (98–272 km) By boat: 42–65 nautical miles (depending on weather conditions)			
Average population size in 2020	2081 inhabitants			
Average GP clinic staff descriptions	Clinics: 1–6 GPs, 2–10 practice staff. There were no additional authorized health care staff working at the clinics.			

* Norway averages 0.8 medical secretaries per GP. Data on nurses in general practice clinics not available.

### Themes

The main themes and subthemes that constitute our findings are presented in Table 4. Table 5 presents quotations illustrative of the subthemes.

**Table 4.** Themes and subthemes

Themes	Subthemes
Theme 1: Vulnerability in rural general practice	Use of locums Work overload Weather and distance to hospital
Theme 2: A wide range of patient safety incidents	Irresponsible practice Lack of follow-up Lack of trust and support
Theme 3: Keeping the clinic safe	Local knowledge Constantly watching out for errors

**Table 5.** Illustrative quotations

Theme and subtheme	Illustrative quotations
**Theme 1** Use of locums Work overload Weather and distance to hospital	‘[The locums have] *mainly been rushing through as many patients as possible to earn maximum money in minimum time, and then they leave. So chronic patients… have been very much left to themselves’.* (G) *‘So we discovered, I guess it was in 2016 or 2017, that several thousand patient notes (and test results) were incomplete and unsigned in our database’* (X) *‘.. in comes a locum GP, who doesn’t know the population, doesn’t know the system, doesn’t know the distance to the hospital, they don’t know how things work. And when they treat the locals for a time, there are bound to be medical errors. They don´t master the language if they’re foreign doctors’.* (G) *‘One locum after another is damaging and expensive. But I don’t mean that a locum GP is an inferior doctor; I reckon he or she may be a terrific doctor. But short-term workers make it difficult to establish a quality healthcare system built on routines and equal treatment for everyone. So not because of the locum* *GP personally, but because you can´t create the environment necessary to combat adverse events and provide continuity and flow’.* (L) *‘For my part, I’ve experienced being so tired at work after way too many consecutive shifts that… I don’t think any adverse events happened, but I thought afterwards: hell, I was really exhausted then. But we’re* *only two GPs so we work long periods of shifts split into two’.* (B) ‘*..and the last of the three patients presented with sepsis, well then I’d simply had enough. Then the [blood-stained, but less] injured patient took too* *much of my attention compared to the one with sepsis.* *So they should have been sent to hospital in a different order’.* (C) *‘During the holiday I was called back, and then the municipality had had no doctor for two days. Ambulance staff had handled the* *acute patients... then I had to work for many consecutive days as the only GP here.* *You limit how many patients you see during the day so you can sleep because of sleepless nights. No one else could take the day shift because I was alone’.* (B) *‘We’re always being told that distance to hospital shouldn’t be part of our assessment. And in theory, that’s all well and good. But distance as a factor in the medical assessment, does sadly play a bigger role than we’d like to acknowledge’.* (N)
**Theme 2** Irresponsible practice Lack of follow-up Lack of trust and support	*‘A patient with diabetes came to the clinic. And the short-term GP… was going to administer insulin to the patient. And if the patient had been given that dose,* *I don´t think he would have survived. The GP said he* *didn´t know how to give insulin’.* (X) *‘… (one foreign doctor) was supposed to be a gynaecologist, but when one of our colleagues here asked him… no he couldn´t do gynaecological examinations. I felt very uneasy about him seeing how little knowledge and language skills he had’.* (J) *‘(the patient was supposed to be) referred to the hospital for an x-ray diagnosis, but in fact (the doctor) didn´t do it. But the patient didn´t know this, so she was waiting around at home for an appointment she never got…this created extra work for the other doctors’. (X)* ‘*First of all, he (medical intern) wanted to give me the important advice never to trust anybody, especially not the colleagues that you think you can trust here. So that’s a problem’.* (K)
**Theme 3** Constantly watching out for errors Local knowledge	*‘I guess I learned what I’ve always said all these years, that you have to constantly watch them… Pay attention so that they (the doctors) do things right, and do what they’re supposed to do’.* (X) *‘A man was calling, and X answered the phone. She’d* *worked at the clinic for about 17–18 years… and the caller was around 50 and asked for a doctor’s appointment, saying there was no rush. But X noticed he wasn’t talking as he usually did and there was something strange about that. So she booked him* *an appointment at the emergency clinic straightaway… and actually he’d had a stroke and was sent straight to hospital’.* (F)

### Theme 1: vulnerability in rural general practice

*Use of locums*. All clinics mentioned the use of locums and frequent turnover of GPs as risk factors for errors and irresponsible practice. The participants described how locums appeared to feel less responsible for following up previous assessments and test results. Locums also lacked local knowledge and participants experienced language barriers.

GPs and practice staff found that patients had difficulty in understanding locums from other parts of Norway or from abroad. Participants described how many locums focused on short-termism and financial gain by seeing as many patients as possible. The use of locums and short-term GPs seemed to affect not only patient treatment but also the local healthcare system as a whole. Interviewees said clearly that the lack of stable personnel reduced healthcare quality by hindering the establishment of routines and making it difficult to correct errors that had occurred.

*Work overload*. The health workers, especially the GPs, found that a major challenge was the frequent shifts at emergency care units, resulting in fatigue, sleep deprivation and cognitive overload.

All participants agreed that this overload was due to staff shortages, and the lack of routines and a buffer in the system. The overload was often amplified when colleagues became ill or merely went on holiday. One GP even remembered being called back several times because the municipality unexpectedly had no doctor.

Practice staff explained how being alone at work led to challenges in priorities, such as leaving the phone ringing while treating a patient. However, the GPs spoke most about being alone and vulnerable when several patients arrived simultaneously.

*Weather and distance to hospital*. During the interviews we heard stories about roads to hospitals being closed due to avalanche risk and dangerous driving conditions. The local health service needed to provide pragmatic care in acute situations when neither aeroplanes nor rescue helicopters could land.

### Theme 2: a wide range of patient safety incidents

The interviewees linked most of the wrong and harmful medical practice to the underlying conditions described in the section above. Participants from clinics with lower turnover and a tolerable workload reported fewer incidents. Examples of drug/alcohol abuse and psychiatric problems were reported in the material. For reasons of anonymity, we cannot describe the most extreme examples of malpractice.

*Irresponsible care*. Some clinics had used locum GPs for many years. Here, we heard stories about unprofessionally high prescription rates of opioids, anxiolytics and excessive sick notes. Several practice staff recalled locums or short-term GPs not taking patients’ problems seriously. One example was a young GP intern who refused to take advice from his supervisor by not admitting a patient to hospital and asked other patients just to google if they had any medical questions or problems. There were also stories about patients not being examined before receiving a diagnosis, or receiving incorrect medication, i.e. not according to Norwegian treatment guidelines, despite having a correct diagnosis.

*Lack of follow-up, trust and support*. Reading and handling medical lab results were situations frequently described as irresponsible. Practice staff could see if the doctor had read and taken action on lab results indicating illness. They told us of some locum GPs who deliberately seemed to choose not to deal with test results or refer patients to specialist care. The GP clinic staff mentioned colleagues and locums they felt they could not trust. They were by no means the majority but were linked to the clinics with greatest turnover. The study participants described how they felt a knot in their stomach in response to such situations.

### Theme 3: keeping the clinic safe

While we heard many examples of malpractice, there were also many cases of practice staff trying to prevent such incidents. Using local knowledge and constantly watching out for errors were the two most prominent ways of trying to keep the clinics safe.

*Local knowledge*. There were stories of patients bypassing appointments with the locum GP just because the medical secretary thought another doctor was better able to treat their condition. Practice staff were crucial in this role since they had usually lived locally for many years. They were vividly portrayed as those with the best knowledge of the local community and skilled in treating common ailments according to Norwegian guidelines.

*Constantly watching out for errors*. The attitude of looking out for errors was mostly described by experienced practice staff usually at clinics with frequent use of locums. We heard several stories about practice staff who had become accustomed to teaching the doctors what to do. Doctors who were judged unsafe or inexperienced needed supervision and sometimes correction to avoid patient safety incidents. The practice staff described how they watched and checked if procedures were followed correctly, if tests were ordered properly, and if the doctor read and acted upon test results. If not, they would not hesitate to intervene, like one medical secretary who stopped an inexperienced short-term GP from giving a patient a potentially lethal dose of insulin.

There were also some non-clinical situations worth noticing. One medical secretary called a locum’s references to check his previous job performances, although the agency vouched for him. When she discovered that he had been repeatedly reported for making serious mistakes, she called the locum doctor agency and told them that this locum was unsatisfactory. He was referred back to the agency.

## Discussion

### Summary of findings

This study generated novel insight into patient safety incidents in rural GP clinics by combining experiences from different types of rural healthcare workers. The findings suggest that system factors like use of locums and work overload are risk factors for irresponsible care and medical errors.

### A fragmented healthcare system

Our findings support the limited evidence that use of locums affects quality and safety of healthcare (25). Most locums in our material were described as good clinicians placed in healthcare organizations with low ability to combine locum work with systematic quality of care. Repeated use of locums creates a disintegrated service where doctors operate for a short time span. This leads to fragmented patient-doctor relationships where traditional relational continuity is difficult to accomplish, aligning with other recent findings in today´s general practice (26). In rural areas with many seniors with complex and chronic conditions, this lack of continuity of care raises particular concern, being likely to increase the risk of patient safety incidents (27), decrease patient satisfaction (28) and even affect mortality (8).

Excessive workload as described here can cause fatigue and impaired psychomotor performance (29). Emotional exhaustion and sleep deprivation have also been demonstrated (30,31). Studies show how these factors predispose clinicians to poor cognitive performance and bias behaviour (32). Work-related problems may thus lead to doctor turnover and discontinuity (33). Discontinuity or ‘gaps in care’ related to failures in communication and care coordination can cause distress and dysfunctional use of healthcare (16). In primary care, discontinuity limits quality initiatives (34) and may have organizational effects on patient safety. When key practitioners leave, they may take with them institutional memory and visions of quality development (34). From a theoretical perspective, the organization suffers by losing stored ‘human capital’, generating human resource costs (35). Depleting social capital by losing staff affects relations and shared trust within the organization (35). The workload challenges call for staff who are present over time, skilled leadership and organization-directed interventions to systematically enable clinical improvements (4).

### Safety support staff

In this study practice staff improved patient safety by providing contextual and experience-based knowledge to locums and GPs. This can be understood as supplying elements of continuity of care (36). Our findings show that by passing on patient information from one locum to the next, ensuring follow-up and providing information on patients’ medical history, family and context, practice staff contribute to patient safety through organizational and informational continuity (36). However, we also presume that important parts of professional and medical information about patients is inaccessible to practice staff.

A minority of the locums described in this study provided irresponsible and unsafe healthcare, and the practice staff acted here more as supervisors. Our study revealed examples of locums with a record of poor work in rural settings. Information on the quality of their previous performance had been readily available to the agencies. Attention should be paid to the information the municipalities receive from locum doctor agencies. To our knowledge, the Norwegian Board of Health Supervision has conducted no inspections of the activity of these agencies. We also question the profitability of the locum doctor markets, which affect the national regular GP scheme, patient-doctor relationships, continuity of quality care and patient safety.

### Strengths and limitations

The strength of this study is primarily the sampling of GPs and practice staff. We included healthcare workers with over 200 years of combined working experience. Combined with incident-focused data, the material gave access to novel and real everyday safety concerns in rural GP clinics. We believe that the field of general practice tends to undervalue and overlook practice staff as a resource for patient safety work and for research. The interviews were conducted in the practice location where the patient safety incident had occurred. The exclusion of locums in our study may have precluded a maximum variation sample. We considered that the experiences of regular workers would highlight the most prominent and typical safety issues in rural general practice, which was the primary interest of the study. The use of locums as an important patient safety issue had, to our knowledge, not previously been highlighted in European patient safety research (37). We share our reflective analysis acknowledging that attention must be paid to the first author’s preunderstanding in interviews and analysis (18), as he is a rural GP. We consider that his background and knowledge of the field were assets in communicating with the participants and for the scope of the study.

Using Skype could perhaps limit interview richness. However, the CIT approach elicits stories rooted in real incidents, and we heard personal and sensitive stories. Interviews were conducted at the local workplace, in small rural communities where ‘everybody knows everybody’. The sensitive topics could make participants experience barriers in reporting some incidents (38). Therefore, participants might have found it easier to discuss safety concerns regarding temporary staff rather than themselves or their co-workers. Nevertheless, five of the 16 interviewees did actually disclose their own personal error incidents.

The data were gathered from rural municipalities. In Norway, however, more than 50% of municipalities are equally rural. Findings from a retrospective study in 2016 showed that 29% of GPs equally distributed in Norway used a locum GP in their practices (10). This number has probably increased. We believe this indicates rural generalizability of our findings and warrants national attention to the patient safety issues presented here.

### Implications for practice

We are worried about patient safety in rural GP clinics with frequent use of locums and work overload. There is an unexploited potential to improve patient safety by offering these clinics a stronger support system and creating new organizational structures that deliver safer care (26,39) The need to recruit and retain skilled healthcare staff is evident (40). Follow-up studies of locums and patient safety seem necessary and important.

## Conclusion

GPs and practice staff experienced patient safety incidents at rural GP clinics. The incidents revealed in this study were diverse. Frequent use of locum GPs and work overload were risk factors for patient safety incidents. Practice staff used various forms of continuity of care to provide patient safety, highlighting strengths but also some major safety concerns in these clinics. Attention is required from local and national healthcare leaders to address patient safety in general practice, especially the consequences of poor continuity and locum profitability. There is a call for further research to understand patient safety challenges in this setting.

## Supplementary Material

cmab064_suppl_Supplementary_Material

## Declaration

Funding: The project was funded by the Norwegian Centre of Rural Medicine, Department of Community Medicine, UiT The Arctic University of Norway. This article’s publication expenses have been covered by a grant from the publication fund of UiT The Arctic University of Norway.

Ethical approval: The study was approved by the Regional Committee for Medical and Health Research Ethics (2016/2314 REK Nord) and conducted according to the guidelines of the Norwegian Centre for Research Data (project #201373), assuring anonymity and data protection.

Patient consent for publication: Not required.

Data availability: Data are available upon reasonable request.
